# Proteolytic shedding of CD46 from human hepatocytes indicates liver stress

**DOI:** 10.1016/j.heliyon.2024.e40841

**Published:** 2024-11-30

**Authors:** Paul Kupke, Jordi Yang Zhou, Gunther Glehr, Paloma Riquelme, Lena Scheibert, Akinbami Adenugba, Hans J. Schlitt, Edward K. Geissler, Jens M. Werner, James A. Hutchinson

**Affiliations:** a Department of Surgery, University Hospital Regensburg, 93053, Regensburg, Germany; b Leibniz Institute for Immunotherapy, 93053, Regensburg, Germany

**Keywords:** Soluble CD46, Steatotic liver disease, Hepatic stress, Liver inflammation

## Abstract

**Background:**

Routine liver function tests capture information about the metabolic and inflammatory condition of the liver, but we lack sensitive biomarkers of early hepatocyte stress. In humans, soluble CD46 (sCD46) levels in blood were recently identified as an accurate biomarker of hepatic steatosis. Here, we explore the diagnostic utility of sCD46 in other liver diseases.

**Methods:**

We developed, optimised and validated an ELISA that facilitates measurements of human sCD46 in plasma, serum and culture supernatants. Then, we analysed mechanisms that lead to the release of sCD46 and identified its role in various hepatic stress conditions.

**Results:**

We discovered that prostaglandin E2 (PGE2) drives upregulation of matrix metalloproteinase (MMP)-1 in fat-loaded hepatocytes, leading to proteolytic shedding of CD46. We further found that sCD46 release was increased by viral, toxic and hypoxic stresses.

**Conclusions:**

sCD46 appears to be a promising biomarker with potential applications in the detection of early liver diseases or monitoring therapeutic responses, which could complement established diagnostic algorithms because sCD46 release is uniquely responsive to hepatocyte stress.

## 1 Introduction

The human liver serves metabolic, detoxifying and immunological functions [1]. Despite its regenerative capacity, liver parenchyma is susceptible to acute and chronic injuries through metabolic, infectious, autoimmune and toxic insults [2,3]. A large panel of biochemical tests are used in daily clinical practice to evaluate different aspects of liver injury [4]. These tests provide information about hepatocyte turnover, inflammation, cell stress and death, metabolic activity and degree of fibrosis, allowing clinicians to distinguish between pathologies and to grade severity [4,5]. To this list, we recently added soluble CD46 (sCD46) release as a marker of hepatocyte stress caused by fat accumulation in steatotic liver disease [6]. In the current study, we aim to refine our clinical interpretation of sCD46 levels through a better understanding of the stimuli and mechanisms leading to sCD46 release.

Elevated blood levels of aspartate transaminase (AST), alanine aminotransferase (ALT) and glutamate dehydrogenase (GLDH) indicate hepatocyte injury, whereas increased levels of γ-glutamyltransferase (γ-GT), alkaline phosphatase (AP) and bilirubin primarily indicate cholestasis [4,5]. Protein synthesis by the liver is measured by production of albumin, coagulation factors and cholinesterase [4,7]. CRP, interleukin (IL)-1β and IL-6 are produced by liver and commonly used as markers of local or systemic inflammation [8]. Beyond these classic markers of liver disease, dynamic tests of liver function, such as lignocaine, methacetin or galactose metabolism tests [[9], [10], [11]], have specialized applications in day-to-day patient management; for instance, these tests can assess physiological liver capacity before major resections [11]. Apart from biochemical tests, biopsy and imaging are routinely used to diagnose or stage hepatological diseases [12]. Clearly missing from this selection of established liver tests is a biomarker of hepatocyte stress that serves as an early indicator of subclinical pathology. Here, we ask whether sCD46 might fill this gap.

We recently identified sCD46 as a reliable biomarker that predicts hepatic steatosis (Grade ≥2) with a correct classification rate (CCR) of 97.8 % (95 % CI [93.5, 100]), positive predictive value (PPV) of 100 % (95 % CI [100, 100]) and negative predictive value (NPV) of 97.6 % (95 % CI [93.0, 100]) [6]. CD46 (or membrane cofactor protein, MCP) is ubiquitously expressed by all human cells except erythrocytes and serves at least four critical functions [13]. First, CD46 regulates complement activation, thereby protecting cells from complement-mediated damage [14]. Second, CD46 is a signal transducer that costimulates CD4^+^ effector T cells to secrete IL-2, IFN-γ, and IL-10 [[15], [16]], as well as promotes IFN-γ-production by CD8^+^ T cells [17]. Third, CD46 serves as a cellular receptor for several viruses, including group B adenoviruses, measles virus and human herpesvirus 6 [[18], [19], [20]]. Fourth, as we recently reported, CD46 expressed by human hepatocytes suppresses the differentiation of IL-4-producing (IL-4^+^) invariant Natural Killer T cells (iNKT) through a presently undescribed cell contact-dependent mechanism [6].

We now know that sCD46 is released from the surface of human hepatocytes after fat loading, and then accumulates in blood [6]. Shedding of sCD46 is mediated by metalloproteinases that are induced by fat exposure [6]. In this work, we establish and validate a sCD46 ELISA to accurately quantify sCD46 concentrations in human blood and cell culture supernatants. We use this assay to investigate various pathological insults, including hypoxic, metabolic, toxic, viral and inflammatory stressors, as stimulators of sCD46 shedding.

## 2 Methods

### 2.1 Study approval and patient cohorts

Samples from two independent patient cohorts were used in this study. Both were part of single-centre, prospective, observational studies in accordance with the Declaration of Helsinki and all other applicable laws and ethics. All patients gave full and informed verbal and written consent to participate. Serum and plasma samples were stored at −80 °C until further analysis. In the first study, 158 patients were included due to planned resection of a liver tumour, which was authorized by the Ethics Committee of the University of Regensburg (votum 13-257-101) and registered at clinicaltrials.gov (NCT04943978). The second study was conducted with 42 patients that presented for antiviral treatment of a chronic hepatitis C virus infection, which was also authorized by the Ethics Committee of the University of Regensburg (votum 12-101-0072).

### 2.2 sCD46 ELISA

A step-by-step protocol for our sCD46 ELISA is provided as supplementary information (Fig. S1). Each incubation is performed at room temperature in the dark. After every step the plate is washed three times by decanting and filling the wells with wash buffer (WA126, R&D systems) using a squirt bottle. First, 100 μl/well of coating antibody (4 μg/ml, MAB2005, R&D systems) is added to an ELISA microplate (DY990, R&D Systems). After incubating at room temperature for 24 h, blocking is performed for 1 h using 300 μl/well reagent diluent (DY995, R&D Systems). Next, 100 μl/well of samples, the corresponding 1:2 serial diluted standards of rhCD46 (2 ng/ml to 31.25 pg/ml, 10256-CD, R&D Systems) and a blank control are added and then incubated for 2 h. Then, 100 μl/well detection antibody (50 ng/ml, BAF2005, R&D Systems) is added followed by another incubation for 2 h. Next, 100 μl/well of Streptavidin-HRP (DY998, R&D Systems) is added followed by 100 μl/well of substrates (DY999, R&D Systems) for 20 and 30 min, respectively. Finally, 50 μl/well stop solution (DY994, R&D Systems) is added without previous washing to terminate the reaction. The plate is then read using a microplate reader set to 450 nm (Varioskan Flash, Thermo Fisher). Final titers are calculated using the in-run standard curve.

### 2.3 Competitive FACS-based sCD46 assay

The performance of our ELISA was compared to a previously described competitive flow cytometry-based assay [6]. Briefly, 3 × 10^5^ CD46-expressing MOLT-4 cells were stained with a PE-conjugated CD46 antibody (REA312, Miltenyi Biotec) after sequential incubation with ViaKr-808 (Beckman Coulter, USA) and 10 % human FcR block (Miltenyi Biotec). The cells were resuspended in a final volume of 100 μl, comprising 0.25 μl of α-CD46-PE mAB and the test sample or a rhCD46 calibration control. The addition of competing rhCD46 leads to a reduced binding of α-CD46-PE mAB to the target cells. Data collection was performed using a CytoFlex LX cytometer and was analysed with Kaluza 2.1 (both from Beckman Coulter). Absolute concentrations were calculated through the calibration controls.

### 2.4 Differentiation and manipulation of HepaRG cells

HepaRG cells (RRID: CVCL_9720, Biopredic International) were cultured for a total of 4 weeks in either 12-well plates, 96-well plates or T75 flasks (all TPP) at 2.5 × 10^4^ cells/cm^2^ in William's E medium supplemented with 10 % HyClone FetalClone II serum, 1 % penicillin/streptomycin, 1 % L-glutamine, 0.023 IE/ml insulin, 4.7 μg/ml hydrocortisone and 80 μg/ml gentamycin. For the last 14 d, 1.8 % DMSO was added to the medium to support differentiation into hepatocyte-like cells. Cells were routinely tested for contamination with mycoplasma.

### 2.5 Stress induction in HepaRG cells

HepaRG cells were fat-loaded (FL) after culture in serum-free medium supplemented with 1 % bovine serum albumin (BSA) for 24 h. The cells were then treated for 24 h with fat-loading medium containing palmitic acid and oleic acid solubilised in isopropanol (ratio 1:2, final concentration 0.5 mM). Vehicle-only control cultures (UL) were treated only with isopropanol. Oil Red O staining (ScienCell) was performed to detect fat-loading according to the manufacturer's instructions (Fig. S2). Using methods established and validated in our laboratory, Hepatitis E virus (HEV) replicating HepaRG cells were generated as previously described [21]. Briefly, differentiated HepaRG were inoculated with HEV supernatants containing 10^7^ to 10^8^ copies/ml of HEV-3c strain 14-16753, which was kindly provided by Prof. Jürgen J. Wenzel (Institute of Clinical Microbiology and Hygiene, University Hospital Regensburg). Viral RNA was detected by RT-PCR as previously described [22]. Hypoxic condition was generated by culturing HepaRG in an atmosphere of 0 % O_2_, 5 % CO_2_ and 95 % N_2_ for 48 h. Heat shock was induced by incubating the HepaRG cell culture plates in a water bath at 45 °C for 30 min. To induce oxidative stress, 250 μM H_2_O_2_ (Roth) was added to the differentiation medium for 24 h. The drug screening was performed by using the Tocriscreen Library of FDA-Approved Compounds (5932, Biotechne). During the fat-loading process, DMSO was reduced to 1.25 % and 10 μM drug was added. For broader titration of prostaglandin E2 (PGE2, Tocris) and TAPI-1 (Cayman Chemical), the same protocol was used with 2 % DMSO.

### 2.6 RNA isolation and qRT-PCR

RNA isolation from HepaRG cells, reverse transcription and qPCR were performed using RNeasy Mini Kit (74106, Qiagen), RNase-Free DNase Set (79254, Qiagen), SuperScript III (18080093, Invitrogen) and QuantiNova Probe RT-PCR Kit (208352, Qiagen) with a LightCycler 480 (Roche). The Quantitect primers (Qiagen) used are listed in Supplementary Table 2. Beta-actin was applied as a housekeeping gene.

### 2.7 MMP1 ELISA

To analyse MMP1 concentrations in the culture supernatants after PGE2 treatment, a commercially available MMP-1 ELISA (R&D Systems) was performed following the manufacturer's instructions.

### 2.8 Statistics

Significance tests were performed in GraphPad Prism v10 as indicated in the figure legends. Where applicable, p-values were adjusted for multiple comparison using the Benjamini-Hochberg method.

## 3 Results

### 3.1 Establishing an ELISA for human sCD46

**Fig. 1:**
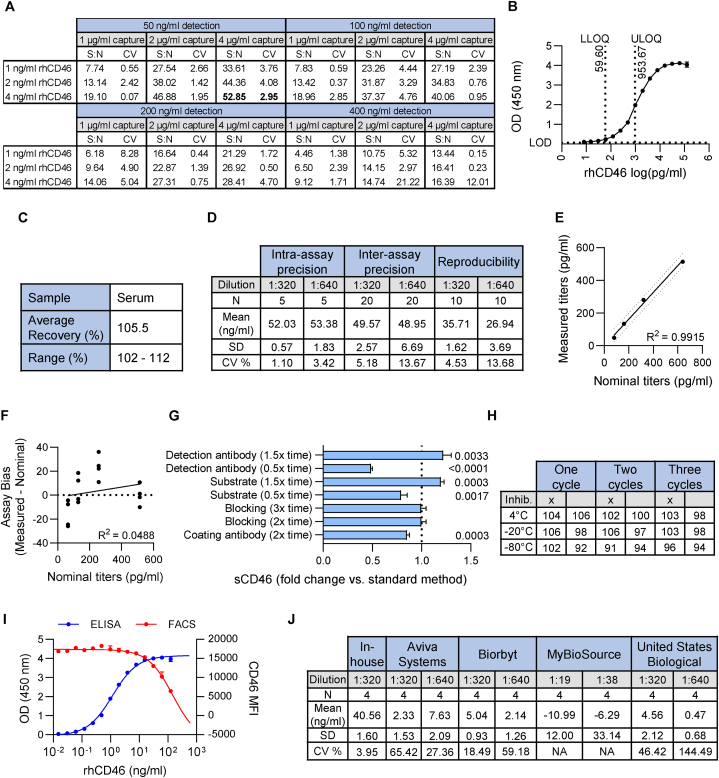
Establishing and validating an ELISA for soluble CD46 (sCD46). **(A)** Signal:noise (S:N) values for different concentrations of CD46 capture and detection antibodies in Reagent Diluent 2 (n = 2). **(B)** Titration curve using recombinant human CD46 (rhCD46) as the analyte. Lower limit of quantification (LLOQ), upper limit of quantification (ULOQ) and limit of detection (LOD) are indicated (n = 3). **(C)** Accuracy (spike recovery) of human serum samples (n = 5). **(D)** Intra-assay precision, inter-assay precision and reproducibility of human serum sample at two different dilution factors. **(E)** Analysis of assay linearity (n = 4, Pearson correlation, indicating 90 % prediction bands). **(F)** Linear regression of the bias plotted against nominal titres (n = 4, Pearson correlation). **(G)** Robustness based on incubation timing (n = 4, one sample *t*-test to a mean of 1). **(H)** Stability of sCD46 (pg/ml) in representative cell culture supernatants over multiple freeze/thaw cycles depending on the storage temperature and the application of a protease inhibitor. **(I)** rhCD46 titration in ELISA and competitive FACS-based assay (n = 3). **(J)** Performance of commercial CD46 ELISA kits in detecting serum sCD46 compared to our in-house ELISA. Samples dilutions were based on manufacturers' recommendations.

Because no commercial assay was available to measure sCD46 after proteolytic release from human cells, we first developed an ELISA. In a cross titration experiment, we determined optimal concentrations for capture and detection antibodies using two different blocking buffers to maximize the signal-to-noise ratio (Fig. 1A, Table S1). Optimal detection conditions were obtained at 4 μg/ml CD46 capture antibody, 50 ng/ml detection antibody and Reagent Diluent 2. These conditions were kept for all further experiments. To establish the range of our assay, we made a 2-fold serial dilution of recombinant human (rh)-sCD46 in triplicate, ranging from 7.45 pg/ml to 122.07 ng/ml. The resulting calibration curve established the range of linearity between 59.60 pg/ml and 953.67 pg/ml (Fig. 1B). By convention, the lower and upper limits of quantification by ELISA are defined by the lowest and highest concentration of analyte returning a coefficient of variation (CV) < 30 % and a backfit (ie. measured/nominal titer x 100) of 80–120 %. For our assay, the lower limit of quantification (LLOQ) was 59.60 pg/ml and the upper limit of quantification (ULOQ) was 953.67 pg/ml (Fig. 1B). The limit of detection (LOD) was 8.95 pg/ml, which was taken as the mean + 3 SD of 12 blank readouts (Fig. 1B).

Because serum components could potentially interfere with assay performance, we tested our ELISA by spiking serum samples with set concentrations of rh-sCD46. By comparing concentrations of sCD46 in unspiked and spiked samples, we determined the assay bias from the nominal and measured values, which was acceptable at < 25 %. Based on 5 serum samples with a mean bias of 5.5 %, our assay returned accurate measurements of sCD46 in human serum samples (Fig. 1C).

### 3.2 Validating an ELISA for human sCD46

To validate our method, we tested its replicability by measuring intra-assay precision, inter-assay precision and reproducibility. To determine intra-assay precision, the concentration of sCD46 was measured five times using human serum samples at two different dilutions with an acceptance criterion of CV < 10 %. The dilution 1:320 returned a CV of 1.10 % and the 1:640 dilution a CV of 3.42 %. Inter-assay precision was tested by repeating the preceding experiment on four consecutive days, giving mean CV values of 5.18 % and 13.67 % at 1:320 and 1:640 dilutions, respectively. Comparing two independent operators, we obtained CV values of 4.53 % at 1:320 dilution and 13.68 % at 1:640 dilution. Hence, our ELISA exhibited good intra-assay and inter-assay precision, and was reproducible under regular laboratory conditions (Fig. 1D).

Next, we assessed the linearity of our sCD46 ELISA. We tested four serial dilutions (1:80, 1:160, 1:320, 1:640) using human serum samples. This showed that measured and nominal values were closely correlated with R^2^ = 0.9915 and gradient = 1.021; hence, our assay showed acceptable linearity (Fig. 1E). To assess whether the matrix complex of Reagent Diluent 2 biased the assay across the dilution range, we plotted residues in a bias-nominal graph, which did not reveal any relevant effect (R^2^ = 0.0488, Fig. 1F). The robustness of our assay was further assessed by varying incubation times in the protocol. Changing the incubation times of coating antibody, analyte or capture antibody resulted in significant deviations (Fig. 1G) whereas doubling or tripling the blocking time did not affect results. Therefore, adherence to the proposed standard operating procedure is essential for inter-experiment comparability (Fig. S1).

Stability of sCD46 in serum samples was tested over multiple freeze-thaw cycles to determine whether storage temperature or use of a protease inhibitor affected our ELISA measurements. Up to 11.3 % degradation of sCD46 signal was observed after 3 freeze-thaw cycles without the addition of a protease inhibitor; therefore, we consider that sCD46 in frozen serum samples is sufficiently stable for measurement using our ELISA for most experimental purposes (Fig. 1H).

### 3.3 Comparing the performance of sCD46 ELISA to other methods

Next, we compared our ELISA assay to a flow cytometry-based assay that was used to measure sCD46 concentrations in our previous work [6]. Our in-house ELISA was clearly superior in ranges below 15 ng/ml (Fig. 1I). Finally, by measuring human serum we compared our assay to four commercially available ready-to-use ELISA kits from MyBioSource (MBS3802163), United States Biological (357260), Biorbyt (orb562879) and Aviva Systems Biology (OKEH00920). All commercial assays were performed according to the manufacturers’ recommendations but they were either unable to detect sCD46 or returned unacceptably high CV values (Fig. 1J).

**Fig. 2:**
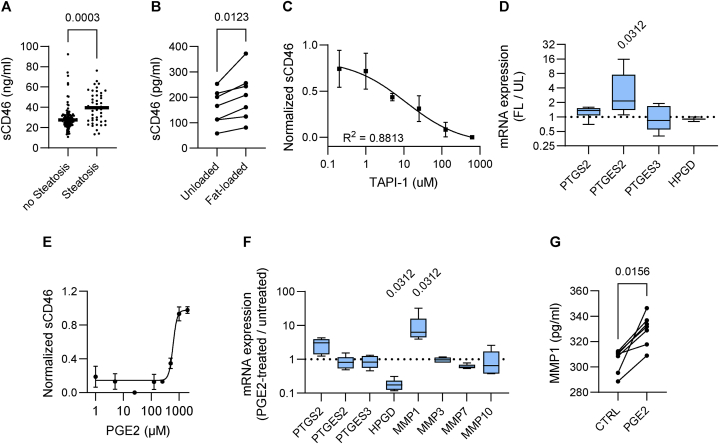
Fat-induced shedding of CD46 involves the prostaglandin E2 (PGE2) pathway and matrix metalloproteinases (MMPs). **(A)** Concentration of serum sCD46 in healthy (n = 112) and steatotic (n = 46) patients (Welch two sample *t*-test). **(B)** Fat-loading (FL) of HepaRG increased the concentration of sCD46 in the culture supernatants compared to unloaded (UL) HepaRG (n = 7, paired two-sample *t*-test). **(C)** The effect of MMP inhibitor TAPI-1 on CD46 shedding measured via ELISA (n = 3, Pearson correlation). **(D)** qPCR results expressed as fold-change in mRNA expression levels of FL/UL HepaRG (n = 6, one-sample Wilcoxon signed-rank test). **(E)** PGE2 treatment induces the shedding of CD46 in FL HepaRG in a dose-dependent manner (n = 3). **(F)** qPCR results expressed as fold-change in gene expression after PGE2 treatment (1 mM) compared to control DMSO (n = 6, Wilcoxon signed-rank test). **(G)** MMP1 ELISA results of FL HepaRG treated with 1 mM PGE2 or control supernatants (n = 7, Wilcoxon signedrank test).

To confirm results from our previous study [6], we used our in-house ELISA to compare serum samples from 46 steatotic patients with those from 112 non-steatotic patients. As expected, we found higher sCD46 levels in samples from steatotic versus non-steatotic patients (Fig. 2A).

### 3.4 Prostaglandin E2 stimulates CD46 shedding

HepaRG cells, a human hepatoma cell line, released more sCD46 into supernatants after fat loading (Fig. 2B). Previously, we showed that CD46 is cleaved from the surface of HepaRG cells by metalloproteinases (MMP) and we could confirm this result by ELISA using a general MMP inhibitor, TAPI (Fig. 2C). Prostaglandins drive MMP expression in many different contexts; therefore, we hypothesized that HepaRG cells upregulate components of the prostaglandin synthesis pathway in response to fat loading, which in turn stimulates MMP expression. *PTGES2* mRNA was significantly induced in fat-loaded HepaRG cells compared to vehicle-only controls (Fig. 2D). We then tested the response of HepaRG cells to exogenous PGE2, finding a threshold-dependent release of sCD46 (Fig. 2E). Treating fat-loaded HepaRG cells with 1 mM PGE2 caused upregulation of *MMP1* mRNA. Interestingly, we observed a significant decrease of *HPGD* mRNA, which is part of the catabolic pathway of prostaglandins (Fig. 2F). The upregulation of *MMP1* mRNA was corroborated by measuring MMP1 concentrations in supernatants by ELISA (Fig. 2G).

### 3.5 sCD46 as a biomarker for non-lethal hepatocyte stress

**Fig. 3:**
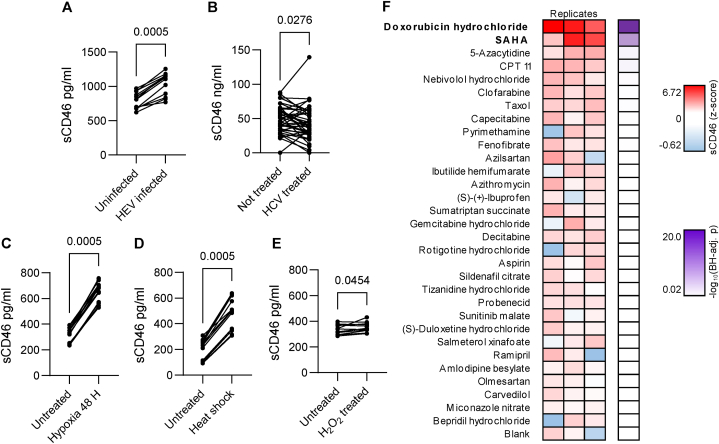
sCD46 as a biomarker for different cellular stresses. **(A)** sCD46 in culture supernatants of hepatitis E virus (HEV) infected HepaRG compared to mock infected controls (n = 12, Wilcoxon signed-rank test). **(B)** sCD46 in plasma of HCV patients before and 12 weeks after successful treatment with direct-acting antivirals (n = 42, Wilcoxon signed-rank test). **(C)** sCD46 in culture supernatants from hypoxic HepaRG cells (1 % O2 for 48 h) compared to normoxic controls (n = 12, Wilcoxon signed-rank test). **(D)** sCD46 in culture supernatants after induction of heat stress (45 °C for 30 min) in HepaRG cells compared to untreated controls (n = 12, Wilcoxon signed-rank test). **(E)** sCD46 in culture supernatants after application of oxidative stress by addition of 250 μM H_2_O_2_ to the medium of HepaRG cultures compared to controls treated with standard medium. (n = 12, Wilcoxon signed-rank test). **(F)** Heat map showing 159 FDA-approved drug substances that upregulated sCD46 levels in HepaRG cell supernatants more than an in-run blank control (n = 3, paired two-sample *t*-test, BH-adj. p-values).

Having confirmed sCD46 shedding is a feature of fat-loaded hepatocytes, we next examined whether other cellular stresses caused the same effect. Infection of HepaRG cells with hepatitis E virus (HEV) led to increased release of sCD46 (Fig. 3A). In a cohort of 42 patients with hepatitis C virus (HCV) infection, we found higher sCD46 levels before initiation of antiviral therapy than after eradication of the virus (Fig. 3B). Further, growth under hypoxic conditions, heat shock and oxidative stress each resulted in increased release of sCD46 from HepaRG cells (Fig. 3C–E).

Because sCD46 was liberated from HepaRG cells in response to diverse, non-lethal cellular stresses, we next asked whether sCD46 might be a useful marker of drug-induced toxicity in hepatocytes. sCD46 release from HepaRG cells was measured after exposure to a panel of 159 FDA-approved drugs. Compared to untreated control cultures, sCD46 release from HepaRG cells was significantly increased by two cytostatic compounds – namely, SAHA and doxorubicin (Fig. 3F).

## 4 Discussion

Here, we extend our original description of sCD46 as a reliable clinical marker for steatotic liver disease by showing that sCD46 release from hepatocytes is stimulated by diverse cellular stresses, which suggests that sCD46 might be a useful marker of liver diseases other than just hepatic steatosis. As a first step in translating our research into an in vitro diagnostic (IVD) assay, we established a robust ELISA that we applied to sCD46 measurement in human plasma samples and cell culture supernatants. Our technical developments allow us to measure sCD46 levels in patients with suspected steatotic liver disease with greater convenience, accuracy and precision than previous methods. Moreover, our discovery that PGE2 induces MMP1 expression to increase sCD46 release from HepaRG cells strengthens the connection between elevated sCD46 levels in blood and liver inflammation.

Unlike other markers of liver injury, sCD46 is not principally released through hepatocyte death; instead, it is liberated by enzymatic cleavage from the surface of hepatocytes in response to cellular stress, which requires an active response of living cells. This implies that sCD46 might be a useful marker of subclinical liver disease, including perhaps drug-induced liver injury. Notably, our screening experiment identified two antineoplastic drugs; hence, we are now planning to test whether sCD46 can be used as an early predictor of chemotherapy-associated liver damage in the context of major liver resections. Such a marker would be valuable in reducing postoperative complications and improving oncological outcomes by optimising the timing of surgery.

In contrast to many routine biochemical markers of liver disease, CD46 is expressed on almost all human cells. Certain diseases, like autoimmune diseases, especially systemic lupus erythematosus and multiple sclerosis [23,24], are associated with increased concentrations of sCD46 indicating that stress-induced shedding of CD46 is not limited to hepatocytes. CD46 expression is also upregulated in malignancies like hepatocellular carcinoma, possibly to escape complement-dependent cytotoxicity, but it remains unknown whether sCD46 titres are correspondingly elevated [25]. sCD46 should therefore always be interpreted in the context of the patient's individual comorbidities.

Knowing about the kinetics of sCD46 release from the hepatocytes and its degradation is key to its interpretation as a clinical biomarker, which we were unable to assess within the scope of this study and therefore represents an important perspective for further investigations. We speculate that sCD46 is generated slowly in patients with hepatic steatosis, but accumulates in blood because clearance is also slow. In other diseases, such as drug-induced liver injury, it is possible that sCD46 is released more rapidly. In liver toxicology, such as acetaminophen poisoning, there is urgent need of new biomarkers that can reliably distinguish patients with self-resolving disturbance of liver parameters from those who progress to acute liver failure [[26], [27], [28]].

In order to optimise sCD46 as a biomarker of liver disease, its mechanism and kinetics of proteolytic shedding from hepatocytes must be compared to those of established liver parameters. γ-GT is largely membrane-bound, so it rises early after hepatobiliary damage [29]. Excessive release of AST compared to ALT indicates the severity of the damage [5], as AST, compared to ALT, also occurs in mitochondria [30]. Further studies have to analyse the kinetics of sCD46 compared to the established repertoire of liver parameters. We anticipate that sCD46 becomes detectable in blood in very early stages of liver disease, possibly even before γ-GT, which is only released through partial disintegration of the hepatocytes’ membrane [29]. Hence, sCD46 could play an important role in the routine diagnosis of immunosuppressed patients following liver transplantation, since current management is largely dependent on quick detection of injury during acute rejection episodes.

Historically, the discriminatory value of many biomarkers has been assessed in steatotic liver disease, especially regarding the progression from benign hepatic steatosis to steatohepatitis, which then poses a significant risk for developing fibrosis. A major challenge here is knowing how specific the signal is to liver inflammation. Promising candidate markers include lipocalin-2 and soluble CD163 (sCD163) that reportedly predict progression to inflammation and fibrosis [[31], [32], [33]]. As sCD46 is superior in detecting hepatic steatosis, possible clinical applications include combining several biomarkers to create a non-invasive composite “signature” to more reliably detect steatotic liver disease and grade risk of progression. Such a panel should allow us to minimize biopsies and imaging studies during routine patient care, which would save costs and reduce the risk of procedural complications.

In summary, sCD46 appears to be a promising biomarker with potential applications in diagnosis of early, subclincal liver diseases. Importantly, because sCD46 release is mechanistically linked to hepatocyte stress, it could enhance the informative value of other liver diagnostic markers that are elevated by very different mechanisms.

## CRediT authorship contribution statement

**Paul Kupke:** Writing – original draft, Visualization, Methodology, Investigation, Formal analysis, Data curation, Conceptualization. **Jordi Yang Zhou:** Writing – original draft, Visualization, Methodology, Investigation, Formal analysis, Data curation, Conceptualization. **Gunther Glehr:** Writing – review & editing, Formal analysis. **Paloma Riquelme:** Writing – review & editing. **Lena Scheibert:** Writing – review & editing. **Akinbami Adenugba:** Investigation. **Hans J. Schlitt:** Writing – review & editing, Resources. **Edward K. Geissler:** Writing – review & editing, Resources. **Jens M. Werner:** Writing – review & editing, Resources, Funding acquisition. **James A. Hutchinson:** Writing – review & editing, Validation, Supervision, Resources, Project administration, Funding acquisition, Formal analysis, Data curation, Conceptualization.

## Ethics declaration

The study complies with all ethics regulations. This study was reviewed and approved by the Ethics Committee of the University of Regensburg with the approval numbers 13-257-101 and 12-101-0072. All study participants provided written informed consent.

## Data availability statement

All data are presented within the manuscript, supplementary materials, accompanying source data file, or are available upon request from the corresponding author.

## Funding

J.Y.Z. was supported by the 10.13039/501100001664Leibniz Institute for Immunotherapy (10.13039/501100015923LIT). G.G. was supported by the 10.13039/100001009Bristol Myers Squibb Foundation for Immuno-Oncology (Award FA-19-64 009). J.M.W. received funding from the Else Kröner Foundation (Award 2015_A10). J.A.H. received funding from the European Union's 10.13039/501100007601Horizon 2020 research and innovation programme (Award 66 860003).

## Declaration of competing interest

University Hospital Regensburg has filed a not yet published European patent application (Registration Nr. 23 183 382.3) for sCD46 as a clinical biomarker of hepatic steatosis. J.A.H. received in-kind support from Beckman Coulter. The authors have no other conflicts of interest to declare.
