# Supplementary material for: Proteolytic shedding of CD46 from human hepatocytes indicates liver stress
Source: Heliyon. 2024 Nov 30;10(23):e40841. doi: 10.1016/j.heliyon.2024.e40841 (PMC11652852; doi:10.1016/j.heliyon.2024.e40841)
Supplement: Multimedia component 1 [file mmc1.pdf]

Figure S1. Sandwich ELISA to Quantify sCD46 (Standard Operating Procedure)

Title	Sandwich ELISA to Quantify sCD46
Purpose	This document describes a method to measure sCD46 in human serum, plasma and cell culture supernatants

Section	Contents
1.	Abbreviations
2.	Reagents
3.	Preparation of reagents
4.	Protocol

1. ABBREVIATIONS

Abbreviation	Definition
DPBS	Dulbecco's phosphate buffered saline

2. REAGENTS

Reagent (R&D systems)	Cat. #	Final dilution/preparation
Human CD46 Antibody	MAB2005	4 µg/ml In DPBS
Clear Polystyrene Microplates	DY990	
Quantikine ELISA Wash Buffer 1	WA126	1:25 in deionized water
Reagent Diluent Concentrate 2	DY995	1:10 in deionized water
Recombinant Human CD46 his-tag Protein	10256-CD	2 ng/ml in reagent diluent/culture medium
Human CD46 Biotinylated Antibody	BAF2005	50 ng/ml in DPBS
Streptavidin-HRP	DY998	1:200 in reagent diluent
Substrate Reagent Pack	DY999	Mix reagent A and B in a 1:1 ratio
Stop Solution 2N Sulfuric Acid	DY994	

3. PREPARATION OF REAGENTS

In-run standard curve	✓
Dilute the CD46 his-tag protein in a 1:2 serial dilution from 2 ng/ml to 31.25 pg/ml in reagent diluent for measuring plasma/serum and for cell culture supernatants in the respective culture medium.	□

4. PROTOCOL

Step	Action	✓
1	Bring all reagents and samples to room temperature. Reagents should be prepared following the manufacturer’s recommendations.	□
2	Add 100 µl/well of CD46 antibody and incubate for 24 h at RT in the dark.	□
3	Wash the plate three times with 400 µl wash buffer using a squirt bottle. After the last washing step, remove remaining wash buffer by decanting and thorough blotting against clean paper towels.	□
4	Add 300 µl/well of reagent diluent and incubate for 1 h at RT in the dark.	□
5	Repeat step 3.	□
6	Add 100 µl/well of samples, the corresponding standards and a blank control and incubate for 2 h at RT in the dark. To reduce technical variability, assay every specimen in duplicates.	□
7	Repeat step 3.	□
8	Add 100 µl/well of CD46 biotinylated antibody and incubate for 2 h at RT in the dark.	□
9	Repeat step 3.	□
10	Add 100 µl/well of streptavidin-HRP and incubate for 20 min at RT in the dark.	□
11	Repeat step 3.	□
12	Add 100 µl/well of substrates and incubate for 30 min at RT in the dark.	□
13	Add 50 µl/well of stop solution.	□
14	Read the plate using a microplate reader set to 450 nm and use the in-run standard curve to calculate the final titers.	□

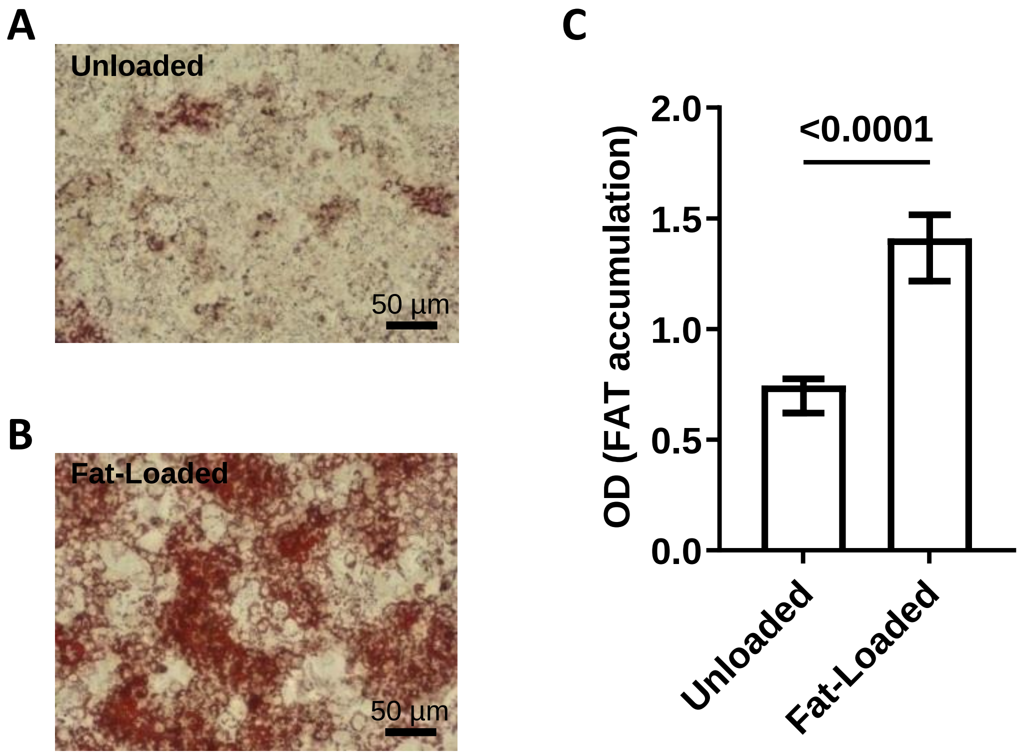

Figure S2. Experimental procedure to verify the successful fat loading of HepaRG cells. **(A, B)** Micrographs of unloaded HepaRG cells and fat-loaded HepaRG cells after Oil Red O staining to measure the accumulation of triglycerides (red). **(C)** Photometric quantification of Oil Red O staining as an indicator of triglycerol accumulation in unloaded and fat-loaded HepaRG cells (n = 20, Wilcoxon signed-rank test).
