# Supplementary material for: Proteolytic shedding of CD46 from human hepatocytes indicates liver stress
Source: Heliyon. 2024 Nov 30;10(23):e40841. doi: 10.1016/j.heliyon.2024.e40841 (PMC11652852; doi:10.1016/j.heliyon.2024.e40841)
Supplement: Multimedia component 2 [file mmc2.pdf]

Supplementary Table 1. ELISA grid experiment. Signal:noise (S:N) values for different concentrations of CD46 capture and detection antibodies in Reagent Diluent 1. (n = 2)

	50 ng/ml detection						100 ng/ml detection					
	1 µg/ml capture		2 µg/ml capture		4 µg/ml capture		1 µg/ml capture		2 µg/ml capture		4 µg/ml capture	
	S:N	CV	S:N	CV	S:N	CV	S:N	CV	S:N	CV	S:N	CV
1 ng/ml rhCD46	3.30	8.36	9.54	3.22	11.53	5.23	3.53	2.65	8.70	4.92	11.00	2.48
2 ng/ml rhCD46	5.56	6.18	15.26	3.72	19.04	4.98	5.62	4.56	14.39	7.01	17.75	0.83
4 ng/ml rhCD46	9.10	9.19	24.72	3.09	29.71	2.46	9.70	4.66	24.15	5.99	29.78	1.98
	200 ng/ml detection						400 ng/ml detection					
	1 µg/ml capture		2 µg/ml capture		4 µg/ml capture		1 µg/ml capture		2 µg/ml capture		4 µg/ml capture	
	S:N	CV	S:N	CV	S:N	CV	S:N	CV	S:N	CV	S:N	CV
1 ng/ml rhCD46	3.15	1.75	7.30	0.31	9.34	0.21	2.41	4.71	5.08	1.03	6.23	1.31
2 ng/ml rhCD46	4.99	1.76	11.99	0.29	15.69	1.55	3.46	0.11	8.46	2.20	10.09	1.50
4 ng/ml rhCD46	7.70	5.56	19.53	0.87	24.90	1.35	5.79	7.90	13.62	1.21	15.53	3.77

Supplementary Table 2. List of qPCR primers.

**Target**	PTGS2	PTGES2	PTGES3
**Cat. No.**	QT00040586	QT00246337	QT00241927

**Target**	HPGD	ACTB	MMP1
**Cat. No.**	QT00013454	QT01680476	QT00014581

**Target**	MMP3	MMP7	MMP10
**Cat. No.**	QT00060025	QT00001456	QT00001470
